# Ex-Vivo Characterization of Bioimpedance Spectroscopy of Normal, Ischemic and Hemorrhagic Rabbit Brain Tissue at Frequencies from 10 Hz to 1 MHz

**DOI:** 10.3390/s16111942

**Published:** 2016-11-18

**Authors:** Lin Yang, Ge Zhang, Jiali Song, Meng Dai, Canhua Xu, Xiuzhen Dong, Feng Fu

**Affiliations:** Department of Biomedical Engineering, Fourth Military Medical University, Xi’an 710032, China; yanglin.0601@163.com (L.Y.); jsj.202@163.com (G.Z.); songjl@fmmu.edu.cn (J.S.); daimeng@fmmu.edu.cn (M.D.); canhuaxu@fmmu.edu.cn (C.X.); dongxiuzhen@fmmu.edu.cn (X.D.)

**Keywords:** stroke, electrical impedance tomography, bioimpedance spectra

## Abstract

Stroke is a severe cerebrovascular disease and is the second greatest cause of death worldwide. Because diagnostic tools (CT and MRI) to detect acute stroke cannot be used until the patient reaches the hospital setting, a portable diagnostic tool is urgently needed. Because biological tissues have different impedance spectra under normal physiological conditions and different pathological states, multi-frequency electrical impedance tomography (MFEIT) can potentially detect stroke. Accurate impedance spectra of normal brain tissue (gray and white matter) and stroke lesions (ischemic and hemorrhagic tissue) are important elements when studying stroke detection with MFEIT. To our knowledge, no study has comprehensively measured the impedance spectra of normal brain tissue and stroke lesions for the whole frequency range of 1 MHz within as short as possible an ex vivo time and using the same animal model. In this study, we established intracerebral hemorrhage and ischemic models in rabbits, then measured and analyzed the impedance spectra of normal brain tissue and stroke lesions ex vivo within 15 min after animal death at 10 Hz to 1 MHz. The results showed that the impedance spectra of stroke lesions significantly differed from those of normal brain tissue; the ratio of change in impedance of ischemic and hemorrhagic tissue with regard to frequency was distinct; and tissue type could be discriminated according to its impedance spectra. These findings further confirm the feasibility of detecting stroke with MFEIT and provide data supporting further study of MFEIT to detect stroke.

## 1. Introduction

Acute stroke is a severe cerebrovascular disease. It can be broadly divided into ischemic stroke caused by cerebral venous thrombosis and hemorrhagic stroke caused by intracerebral hemorrhage or subarachnoid hemorrhage [1]. Stroke is characterized by high morbidity and mortality and is the second greatest cause of death worldwide after ischemic heart disease [2]. The prognosis of stroke patients can be significantly improved if prompt intervention by clinical staff is given. However, two different types of stroke patients require distinct treatment. Ischemic patients are given tissue plasminogen activator, a thrombolytic agent, within 3–4.5 h [3], while hemorrhagic stroke patients are treated with timely surgery. Because thrombolytic therapy makes hemorrhagic stroke worse, before treatment, prompt brain imaging is needed to discriminate ischemic from hemorrhagic stroke. Current diagnostic tools used to detect stroke, such as CT and MRI, are not accessible until patients arrive at a hospital. This means that only 2.5%–6% of 87% of ischemic strokes are treated in time [4]. Consequently, a portable and inexpensive diagnostic tool to detect stroke quickly, which can be used in primary healthcare units and ambulances, is urgently needed. Because biological tissues have different impedance characteristics under normal physiological conditions and different pathological states [5,6,7,8], impedance methods are proposed to detect stroke [9,10,11,12,13,14,15,16,17,18,19,20,21,22,23].

According to the principle of impedance methods to detect stroke, these methods can be mainly divided into three categories. First, based on the fact that the impedance of the whole brain is different before and after stroke occurrence, it is expected to detect stroke by monitoring the dynamic impedance change of the whole brain during stroke [9,10,11,12,13,14,15]. In this method, the impedance before stroke needs to be measured to serve as a reference [16]. Second, because the impedance distribution of both brain hemispheres of normal human is symmetrical, whereas it is otherwise for a stroke patient, it is possible to detect stroke by comparing whether the impedance distribution is symmetrical for the hemispheres in a subject’s head [17,18,19,20]. This method requires that the shape of the subject’s two hemispheres should be basically symmetrical and that the electrodes must be strictly placed [18]. Third, since normal brain tissues and stroke lesions have different spectra, the impedance distribution inside the subject’s head can be reconstructed in order to detect stroke. This is realized by simultaneously injecting currents at multiple frequencies and measuring the corresponding boundary voltages through surface electrodes, namely multi-frequency electrical impedance tomography (MFEIT). Stroke lesions can be identified by their specific impedance spectra. MFEIT does not require reference data in another time point [21], the basic symmetry of both hemispheres in the subject’s head or the strict electrode placement. Thus, MFEIT is a medical imaging tool that could theoretically detect stroke quickly [22,23].

Accurate impedance spectra of normal brain tissue (gray and white matter) and stroke lesions (ischemic and hemorrhagic tissue), especially when obtained from the same animal (thus allowing comparability between different tissues), are important elements when studying stroke detection with MFEIT. Several studies have investigated the impedance of normal brain tissue and stroke lesions. Surowiec et al. measured the impedance of bovine gray and white matter in the frequency range from 20 kHz–100 MHz [24]; Stoy et al. measured the impedance of dog brain tissue at room and body temperature from 100 kHz–100 MHz [25]; Gabriel et al. reported the impedance of bovine brain tissue from 20 Hz–20 GHz, up to 2 h after excision [6]; while other studies reported the measurement of the impedance of brain tissue at several frequencies [26,27]. However, these studies carried out these measurements only in normal brain tissue and not in stroke lesions. Surowiec et al. and Stoy et al. did not measure impedance below 20 kHz (whereas a frequency range of 1 MHz is often used to improve the efficiency of MFEIT in stroke detection [20,28]), and the long ex vivo time may have affected the measurement by Gabriel et al. [25,29].

As for ischemic tissue, Wu et al. compared the impedance of normal brain tissue and ischemic tissue in rabbits within the 1 Hz–1 MHz range [30,31]; Ranck et al. measured the ischemic tissue of rabbits several hours after death [26]. However, these studies did not control for the ex vivo time (in the study by Wu et al., the ex vivo time was not stated). In the case of hemorrhagic tissue (which involves bleeding within the brain tissue itself), Ur et al. compared the impedance of whole blood and clotted blood at a single frequency [32]; Lei et al. used a microfluidic chip to measure the impedance of whole blood and clotted blood from 200 Hz–10 kHz [33]; and Dowrick et al. measured clotted blood coagulating at 4 °C [34], while heparinized blood was measured in other studies [35]. However, these blood samples were not taken from hemorrhagic tissue due to stroke in either animals or humans. In conclusion, to our knowledge, no study has carried out a comprehensive measurement and analysis of impedance spectra of normal, hemorrhagic and ischemic brain tissue for the whole frequency range of 1 MHz within as short as possible an ex vivo time and using the same animal model.

In this study, we established intracerebral hemorrhage and ischemic models in rabbits. Then, we measured the impedance spectra of white matter, gray matter, ischemic and hemorrhagic tissue within 15 min after animal death at frequencies from 10 Hz–1 MHz. We analyzed the feasibility of using MFEIT to detect stroke with regard to the impedance spectra of tissues by comparing the difference in impedance spectra between normal tissue and stroke lesions and the difference in the ratio of change of impedance between hemorrhagic and ischemic tissue with regard to frequency. We also investigated the relationship between the specificity of the tissue impedance spectrum and the type of tissue using partial least squares discriminant analysis (PLS-DA).

## 2. Materials and Methods

### 2.1. Animal Preparation

All experiments in this study were approved by the Institutional Animal Care and Use Committees of the Fourth Military Medical University, Xi’an, Shaanxi, China.

Twenty-two New Zealand rabbits (2 ± 0.5 kg) obtained from the experimental animal center were divided into two groups, 11 in the intracerebral hemorrhage model and 11 in the ischemic model, respectively. Rabbits fasted for 4 h and were not allowed to drink water 2 h before the experimental procedures. They were initially anesthetized via intraperitoneal injection with 1.5% pentobarbital (2 mL/kg). After sedation, 3% pentobarbital (1 mL/kg) was injected into an ear vein to achieve a deep surgical level of anesthesia. Body temperature was measured using a rectal thermistor probe and maintained at 39.5 ± 0.5 °C using a warm-water blanket. Rabbits were placed in a stereotactic frame in the prone position employing raised eye and ear bars. An area of about 10 cm^2^ was removed from each rabbit’s ear; using a scalpel, a vertical incision of about 3 cm in length was made 0.5 cm behind the line connecting the two otic edges. The periosteum within the visual field was also removed.

### 2.2. Surgery

Given that stroke mainly consists of local hemorrhage and ischemia, we established localized intracerebral hemorrhage and ischemic models, respectively.

#### 2.2.1. Intracerebral Hemorrhage Model

**Figure 1:**
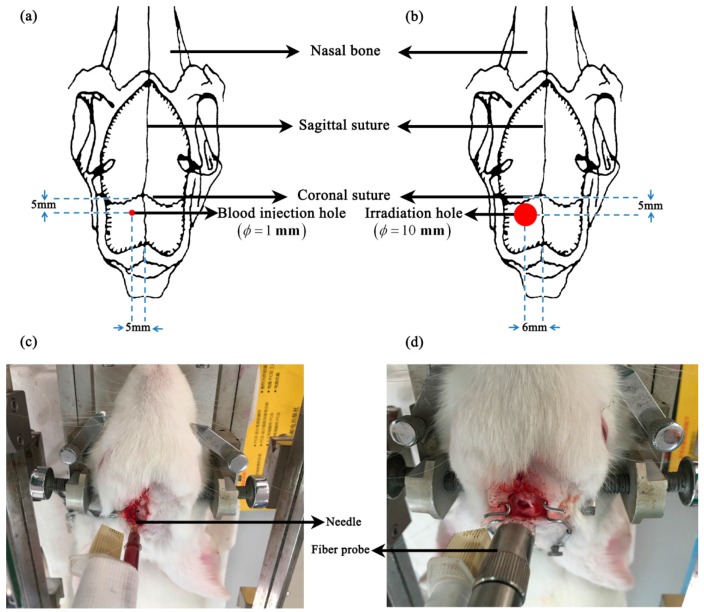
Establishment of the intracerebral hemorrhage and ischemic models. (**a**,**c**) The hemorrhagic model obtained with the autologous blood injection method. (**b**,**d**) The ischemic model obtained with the photothrombotic method.

Autologous blood injection was adopted to produce the intracerebral hemorrhage model. To prepare to inject blood (Figure 1a,c), a hole 1 mm in diameter was carefully drilled 5 mm anterior to the coronal suture and 5 mm to the left of the sagittal suture using a dental drill and just touching the dura. Next, 1 mL of blood was collected from the heart, and 0.35 mL of blood were aspirated into a 1-mL syringe. The syringe was fixed to the stereotactic frame, and the needle was inserted into the brain via the hole to a depth of 11 mm to ensure the correct location of the blood injection at the brain parenchyma, according to the anatomy of brain. Then, the blood was slowly injected within 40 s. To prevent the blood from refluxing through the hole, the needle was left in the brain 10 min after completion of the injection. Then, the needle was slowly withdrawn; the hole was sealed with bone wax; and the wound was sutured. After 30 min, the rabbit was sacrificed by administering a pentobarbital overdose. The rabbit skull was removed, and the whole brain was taken out to measure brain tissues (10 rabbits) or immerse in formalin overnight for 24 h (1 rabbit). The measured tissues included the hemorrhagic tissue (referring to the clotted blood) and white matter tissue on the contralateral side of hemorrhagic tissue.

#### 2.2.2. Ischemic Model

The photothrombotic stroke method was used to establish the ischemic model [36]. Using a dental drill with a 10-mm diameter, a hole was drilled 5 mm anterior to the coronal suture and 6 mm to the left of the sagittal suture. The hole penetrated the outer plate and the diploë, touching the inner plate. A gelatin sponge was used to stop blood flow and clean the visual field (Figure 1b,d); 3.5% Rose Bengal (Sigma-Aldrich Corporation, St. Louis, MO, USA) was injected into the ear vein (1.5 mL/kg). When the rabbit eyelid turned rose red (approximately 10 min after injecting the Rose Bengal dye), the hole was continuously irradiated with a 1 cm in diameter green cold light (wavelength 540 mm, intensity 600 mW/cm^2^) from an LG150B-type cold light source (Photo-machine Technological Exploration Corporation, Shanxi, China) for 40 min and then the wound was sutured. After 40 min, the rabbit was sacrificed by administering a pentobarbital overdose, and the whole brain was removed to measure the tissues (10 rabbits) or immerse in formalin overnight for 24 h (1 rabbit). The measured tissues included ischemic tissue and gray matter on the contralateral side of the ischemic tissue (the ischemia obtained by photothrombotic method mainly occurs in gray matter).

### 2.3. Tissue Impedance Spectra and Electrical Property Analysis

#### 2.3.1. Measurement of Tissue Impedance Spectra

**Figure 2:**
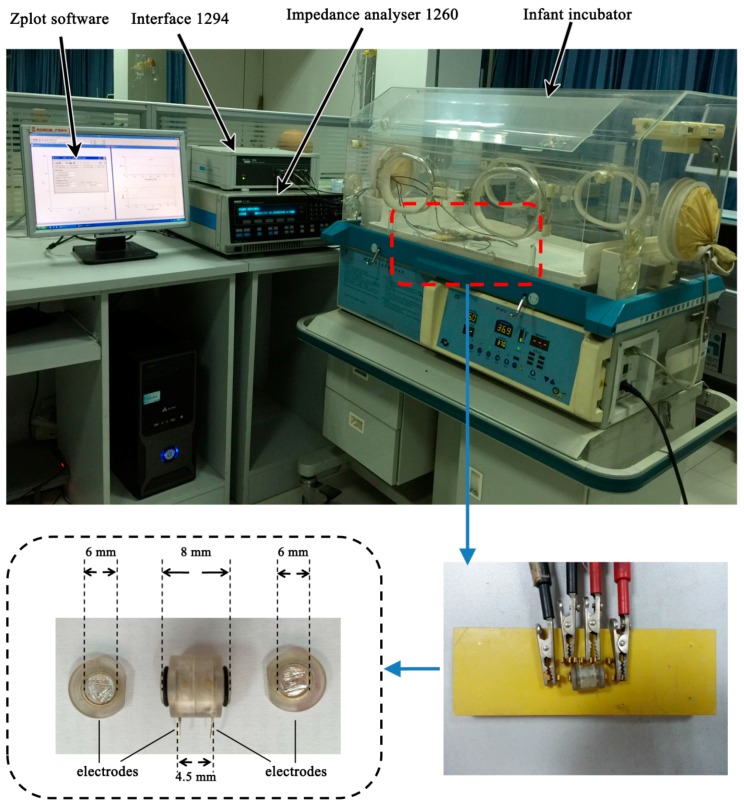
Impedance spectra measurement system including Solartron 1260 impedance/gain-phase analyzer with a 1294A interface and one of the measuring boxes.

After the brain was removed, the gray matter, white matter and stroke lesions (hemorrhagic or ischemic tissue) were separated. To shorten the ex vivo time as much as possible, two experimenters simultaneously loaded the normal tissue or stroke lesions into two measuring boxes (Figure 2). Each measuring box had a cylindrical cavity 6 mm in diameter and 8 mm in length. The tissue was trimmed before being loaded into the measuring box. The purpose of trimming tissue is to shape it so that it matches the space in the measuring box, thereby ensuring the accuracy of the measurement. The measuring box had four silver electrodes; two ring electrodes with a diameter of 6 mm were located at both ends of the measuring box; and the other two ring electrodes in the middle at an interval of 4.5 mm. In this study, the four-electrode technique was used to reduce the effect of electrode and contact impedance [37]. The electrodes at both ends served as exciting electrodes and the electrodes in the middle as measuring electrodes (Figure 2). After the tissues were loaded, the measuring box was connected to the Solartron 1260 impedance/gain-phase analyzer (Solartron Analytical, Farnborough, UK) with a 1294A impedance interface system for measurement, controlled by the Zplot software (Scribner Associates Inc., Southern Pines, NC, USA). A 0.2 mA AC (r.m.s.) signal was used across two exciting electrodes while sweeping the frequencies from 10 Hz–1 MHz in 51 steps. The voltage between the two measuring electrodes was measured, and the impedance was calculated. An infant incubator (Daiwei, Ningbo, China) was used to maintain the temperature at 37 °C, with 60% humidity. In every experiment in this study, when measurement of a tissue type was completed, the measuring box was replaced immediately with another to ensure that all measurements were completed within 15 min.

#### 2.3.2. Saline Solution Control

A 0.03 mol/L saline solution was injected into the measuring box, and its impedance was recorded as the control before each measurement. Peyman et al. obtained the theoretical conductivity of 0.03 mol/L saline solution [38], which is 0.3 S/m for all frequencies.

#### 2.3.3. Properties of Tissue Impedance Spectra

We obtained the tissue impedance (*Z*) with the Solartron 1260 system. If tissue impedance is equivalent to a parallel capacitance and conductance circuit [29], tissue impedance is given by:

(1) $$Z=Z_{real}+jZ_{imag}=\frac{G}{G^{2}+\omega^{2}C^{2}}-j\;\frac{\omega C}{G^{2}+\omega^{2}C^{2}}$$

(2) $$G=\sigma \frac{S}{l}$$

(3) $$C=\varepsilon \frac{S}{l}$$

where $Z_{real}$ is the real part of *Z*; $Z_{imag}$ is the imaginary part of *Z*; *j* is the unit imaginary number, $j^{2}=-1$; *C* is the equivalent capacitance; *G* is the equivalent conductance; *ω* is the angular frequency, ω=2π⋅f; σ is the conductivity; *ε* is the dielectric permittivity.

According to the principle of measurement, the conductivity and dielectric permittivity of tissue can be calculated as follows:

(4) $$\sigma =\frac{Z_{real}\cdot l}{(Z_{real}^{2}+Z_{imag}^{2})\cdot S}$$

(5) $$\varepsilon =\frac{-Z_{imag}\cdot l}{\omega \cdot S\cdot (Z_{real}^{2}+Z_{imag}^{2})}$$

where *S* is the cross-sectional area of the cylindrical cavity of the measuring box and *l* is the length of the two measuring electrodes.

As a result, real and imaginary tissue impedance can be denoted as following the formats relating to the size of the measuring box, conductivity and permittivity:

(6) $$Z_{real}=\frac{\sigma }{\sigma^{2}+{\left(\omega \varepsilon \right)}^{2}}\cdot \frac{l}{S}$$

(7) $$Z_{imag}=\frac{-\omega \varepsilon }{\sigma^{2}+{\left(\omega \varepsilon \right)}^{2}}\cdot \frac{l}{S}$$

To directly compare the impedance spectra of different tissues, real and imaginary normalized impedance can be obtained if *S* = 1 cm^2^ and *l* = 1 cm.

In this study, we used *σ*, *ωε* (permittivity), $Z_{real}$ and $Z_{imag}$ to display the impedance spectra of all tissues.

### 2.4. Histopathology

To assess the histopathological change in stroke lesions, 1 cm in diameter sections of white and gray matter, and hemorrhagic and ischemic tissue were removed from the rabbit brain after animal death. Samples were fixed in 10% formalin for 24 h. Then, samples were cut into 3 mm-thick slices for hematoxylin and eosin staining and were reviewed by one pathologist (HMR) under an optical microscope.

### 2.5. Data Analysis

We used IBM SPSS Statistics for Windows, Version 20.0 (IBM Corporation, Armonk, NY, USA) to carry out the statistical analysis. The comparison of tissue impedance at different frequencies was performed with a paired samples *t*-test.

To detect stroke with MFEIT, the first step is to discriminate stroke lesions from normal brain tissue. We used the impedance spectrum variation index (*SVI*)—$SVI^{\sigma }$ and $SVI^{\left(\omega \varepsilon \right)}$—to compare the difference in impedance spectra between stroke lesions and normal brain tissue (see Equations (8) and (9)). In consideration of the frequency dependence of tissue impedance, we used conductivity and permittivity to study the change in impedance:

(8) $$SVI^{\sigma }=\frac{\sigma_{f}^{s}-\sigma_{f}^{n}}{\sigma_{f}^{n}}$$

(9) $$SVI^{\left(\omega \varepsilon \right)}=\frac{2\pi f\varepsilon_{f}^{s}-2\pi \cdot f\cdot \varepsilon_{f}^{n}}{2\pi \cdot f\cdot \varepsilon_{f}^{n}}$$

where $\sigma_{f}^{s}$ and $\sigma_{f}^{n}$ are the conductivity of stroke lesions and normal brain tissue at *f*, respectively, and $\varepsilon_{f}^{s}$ and $\varepsilon_{f}^{n}$ are the permittivity of stroke lesions and normal brain tissue at *f*, respectively.

Second, at the early stages of acute stroke, it is important to differentiate stroke tissue from normal brain tissue, but also discriminate the type of stroke. The difference in the ratio of change in impedance with frequency between hemorrhagic and ischemic tissue may be useful to discriminate stroke type. We divided the frequency range into three parts to analyze the ratio of change in impedance of hemorrhagic and ischemic tissue, respectively: the low-frequency range (10 Hz–1 kHz; 10 Hz is the reference frequency); the middle-frequency range (1 kHz–100 kHz; 1 kHz is the reference frequency); and the high-frequency range (100 kHz–1 MHz; 100 kHz is the reference frequency). For each frequency range, the conductivity or permittivity at the reference frequency was compared with the other frequencies, as denoted by $\left(\sigma_{f}-\sigma_{f_{ref}}\right)/\sigma_{f_{ref}}$ and $\left(2\pi f\varepsilon_{f}-2\pi f_{ref}\varepsilon_{f_{ref}}\right)/2\pi f_{ref}\varepsilon_{f_{ref}}$.

To study the relationship between tissue type and tissue impedance spectrum, we used PLS-DA to evaluate the statistical distribution of tissue impedance spectra, namely, discriminating each type of tissue by its impedance spectrum. PLS-DA has been widely used in the analysis of spectral data [39,40] and when analyzing problems related to classification or clustering [41,42]. The Unscrambler 9.7 software from Camo Inc. was used to perform PLS-DA.

## 3. Results

**Figure 3:**
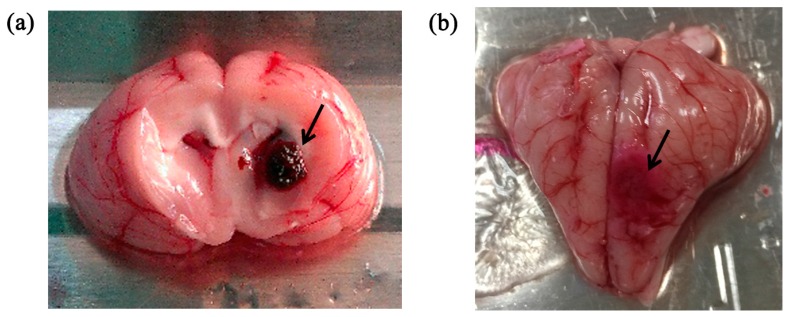
(**a**) The intracerebral hemorrhage model; (**b**) The ischemic model. The black arrow indicates the location of the stroke lesion.

In all experiments, animals retained stable body temperature and respiration. While establishing the intracerebral hemorrhage model in two animals, blood broke into the ventricle; therefore, we added two more animals. Forty sets of data were obtained, 10 sets of data for gray matter, 10 for white matter, 10 for hemorrhagic and 10 for ischemic tissue, respectively. The hemorrhagic (clotted blood was approximately 280 ± 20 mm^3^) and ischemic (ischemic tissue was 4.5 ± 0.25-mm in diameter and 2.9 ± 0.1-mm in depth) models are shown in Figure 3.

### 3.1. Measurement of the Impedance Spectra of Normal Brain Tissue and Stroke Lesions

The conductivity of the 0.03 mol/L saline solution remained at 0.287 S/m across all frequencies. This validated the reliability of our measurements.

**Figure 4:**
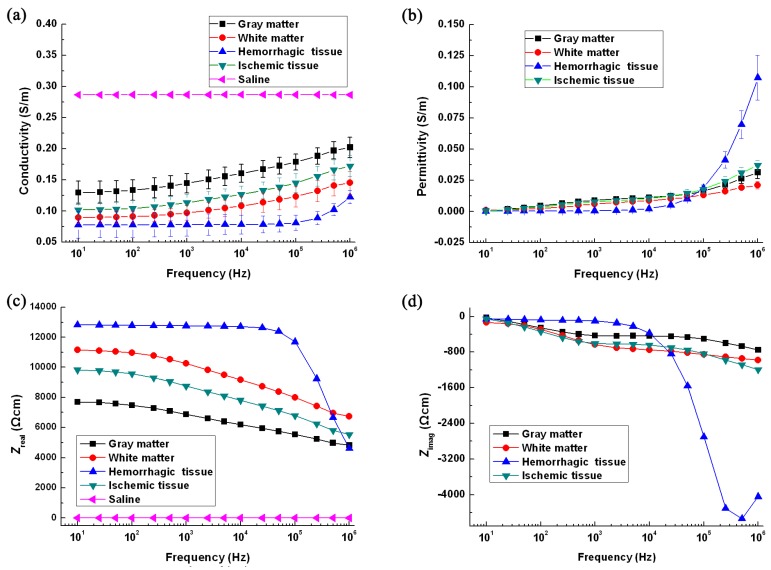
Results of impedance measurement. (**a**–**d**) The conductivity, additivity and the real and imaginary part of normalized impedance, respectively.

Figure 4a,b shows the conductivity and permittivity of all tissues, respectively. In terms of change in conductivity and permittivity, the conductivity of white matter, gray matter and ischemic tissue increased with frequency (*p* < 0.05; all tissues at 10 Hz and 1 MHz), while the conductivity of hemorrhagic tissue remained constant below 100 kHz and then increased within the 100 kHz–1 MHz range (*p* < 0.05 at 100 kHz and 1 MHz). The change in permittivity was similar to the change in conductivity. With regard to the magnitude of conductivity and permittivity, the conductivity of hemorrhagic tissue was smaller than that recorded for the other tissues. The difference in permittivity in ischemic tissue, and in white and gray matter, was small below 100 kHz, and the permittivity of hemorrhagic tissue was significantly different from the other three tissue types within the whole frequency range.

Figure 4c,d shows the real and imaginary part of normalized impedance. The real part of ischemic tissue, and white and gray matter, decreased, and there was a local extreme point near 1 kHz. According to dispersion theory [43], 1 kHz should correspond to the characteristic frequency of initial dispersion (α-dispersion). For hemorrhagic tissue, this same phenomenon occurred in the frequency range between 100 kHz and 1 MHz, indicating that the initial dispersion of hemorrhagic tissue might occur within this frequency range.

### 3.2. Difference in Impedance Spectra between Normal Brain Tissue and Stroke Lesions

**Figure 5:**
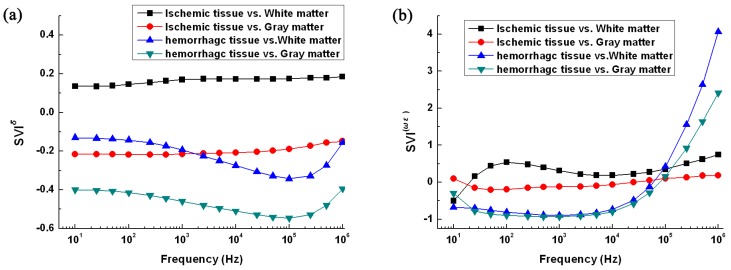
Difference in conductivity (**a**) and permittivity (**b**) between normal brain tissue and stroke lesions at each frequency within 1 MHz. A vs. B (A and B are two kinds of tissues) represents the relative difference between A and B, namely, *SVI* = (A − B)/B.

Figure 5a shows that the difference in conductivity between ischemic and normal tissue changed slowly with frequency and remained approximately at 0.2 times. The differences between hemorrhagic and normal tissue changed significantly with frequency. At high frequencies (>100 kHz), the difference could be up to 0.6- and 0.3-times greater compared with gray and white matter, respectively.

Over the whole frequency range (Figure 5b), the difference in permittivity between normal tissue and stroke lesions was greater than the difference in conductivity. The difference in permittivity between ischemic tissue and white matter increased with frequency below 200 Hz before decreasing with frequency above 200 Hz. The difference in permittivity between ischemic tissue and white matter changed slowly with frequency except below 100 Hz. The difference in permittivity between hemorrhagic and normal tissue was up to two-times greater above 100 kHz.

### 3.3. Difference in the Ratio of Change in Impedance with Frequency between Ischemic and Hemorrhagic Tissue

**Figure 6:**
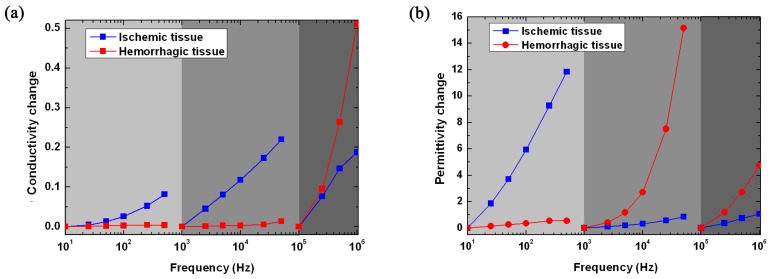
Ratio of change in conductivity (**a**) and permittivity (**b**) of ischemic and hemorrhagic tissue with regard to the low-frequency (10 Hz–1 kHz; 10 Hz is the reference frequency), middle-frequency (1 kHz–100 kHz; 1 kHz is the reference frequency) and high-frequency (100 kHz–1 MHz; 100 kHz is the reference frequency) ranges.

Figure 6a shows that the ratio of change in conductivity of ischemic tissue nearly linearly increased with frequency (logarithmic frequency shown in the figure) in the low- and middle-frequency ranges. The maximum increments in these two frequency ranges were approximately 10% and 20%, respectively. The ratio of change in conductivity of hemorrhagic tissue remained almost zero in the low- and middle-frequency ranges. However, in the high-frequency range, the conductivity of stroke lesions increased with frequency. In brief, the difference in the ratio of change in conductivity between ischemic and hemorrhagic tissue was noticeable in the low and middle frequencies.

Figure 6b shows that the ratio of change in permittivity of ischemic tissue was significantly greater than that of hemorrhagic tissue, up to 10-times greater in the low-frequency range; in the middle and high frequencies, the ratio of change in the hemorrhagic tissue was significantly greater than that of the ischemic tissue. Thus, the difference in the ratio of change in permittivity between stroke lesions was distinct.

### 3.4. Using Impedance Spectra to Discriminate Tissue Type

Figure 4a,b shows that the conductivity and permittivity spectra of the four kinds of tissues were different. To further analyze the relationship between the specificity of the impedance spectra and tissue type, PLS-DA was used to discriminate tissue type by means of impedance spectra.

In the PLS-DA model, the independent variable, X, consisted of the conductivity or permittivity of all tissues at each frequency (51 frequencies from 10 Hz–1 MHz; X dimension: 40:51), while the dependent variable, Y, consisted of the 40-set sample of gray matter (10), white matter (10), hemorrhagic (10) and ischemic (10) tissue, which were assigned to 1–1.1 (0.01 increments), 2–2.1 (0.01 increments), 3–3.1 (0.01 increments) and 4–4.1 (0.01 increments), respectively.

**Figure 7:**
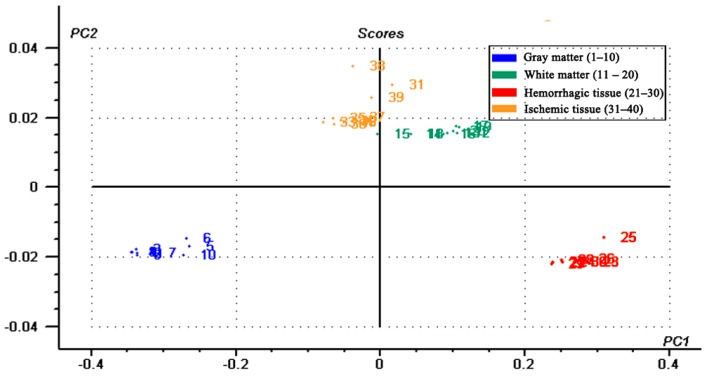
Principal component analysis score plot from the partial least squares discriminant analysis showing the first two principal components.

**Figure 8:**
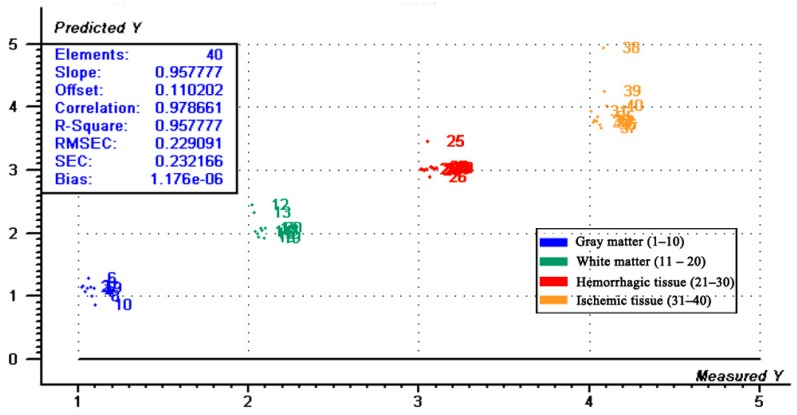
Regression plot of the data from the partial least squares discriminant analysis showing the first two principal components (see Figure 7).

The PLS-DA results for conductivity are shown in Figure 7 and Figure 8. As shown by the principal component analysis (PCA) score plot, all measurements fell within their own unique tissue-specific area, indicating that four kinds of tissues could be clearly distinguished. The regression plot showed four kinds of tissue located in four ranges of predicted values. In the plot, all data associated with gray matter, white matter, hemorrhagic and ischemic tissue had a predicted Y value <1.5, <2.7, <3.7 and >3.7, respectively. The results of the PLS-DA for permittivity were similar to those for conductivity and are not shown.

### 3.5. Histopathology

**Figure 9:**
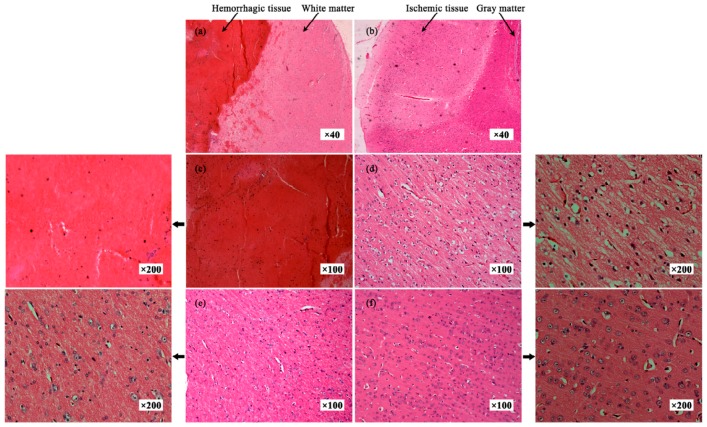
Hematoxylin and eosin-stained images of normal brain tissue and stroke lesions. (**a**,**c**,**e**) The hemorrhagic tissue and surrounding normal white matter and hemorrhagic tissue and white matter, respectively. (**b**,**d**,**f**) The ischemic tissue and surrounding normal gray matter and ischemic tissue and gray matter, respectively.

Figure 9a shows the hemorrhagic tissue (clotted blood) and surrounding normal white matter. A large number of red blood cells can be seen (Figure 9c). Normal white matter cells are arranged neatly and tightly (Figure 9e). Figure 9b shows the ischemic tissue and surrounding normal white matter. Figure 9d shows that the cells of ischemic tissue have undergone morphological changes, and some cells display swelling, indicating edema as a result of ischemia. As shown by Figure 9c–f, the structures of the cells of stroke lesions were different from normal tissue and hemorrhagic tissue was different from ischemic tissue.

## 4. Discussion

### 4.1. Summary of Experimental Results and Comparison with Previous Studies

The significant difference between our study and previous ones is that we comprehensively measured the impedance spectra of normal brain tissue and hemorrhagic and ischemic tissue, from 10 Hz–1 MHz, and analyzed them within 15 min after animal death.

In the case of normal brain tissue, white matter impedance was greater than gray matter impedance over the whole frequency range (Figure 4), which largely agrees with previous studies [6,26]. However, the change in impedance in normal tissue in this study is different from previous studies. Gabriel et al. reported that the conductivity of gray matter significantly increased below 100 Hz, with a change of about 18% [6], while the change was approximately 4% in our study. This was probably because, in the study by Gabriel et al., the measurement was performed up to 2 h after animal death. In fact, after animal death, ion concentration in tissues and changes in cell membrane structure affect tissue impedance. To reduce the effect of ex vivo time on tissue impedance, all measurements in our study were made within 15 min after animal death.

The change in impedance in ischemic tissue was about 40% from 10 Hz–1 MHz. Ranck et al. reported that the change within the 5 Hz–50 kHz could be as great as 200% [26], but the measurement was carried out ex vivo several hours after animal death. We also found that the impedance of ischemic tissues was greater than that of gray matter, especially at low frequencies (<1 kHz). The most plausible explanation for this phenomenon is that the pathological changes occurring in tissue after ischemic stroke lead to a change in impedance. When ischemia is caused by a reduction in blood flow, the ischemic cells switch from aerobic to anaerobic metabolism to overcome the lack of oxygen. The osmotically-active products produced by anaerobic metabolism accumulate in cells, resulting in increased intracellular osmotic pressure and ion concentration. To balance the osmotic pressure inside and outside the cell, water flows from the extracellular to the intracellular space, causing the cell to swell (Figure 9c) [44]. As a result, the extracellular space is reduced, causing an increase in tissue impedance. Because current at low frequencies flows through the extracellular space, a change in tissue impedance at low frequencies is most obvious (Figure 5). Additionally, we found that the difference in the imaginary part of impedance between normal tissue and stroke lesions was greater than the real part (Figure 5), so the imaginary part contained the important information needed for stroke detection, although the magnitude of the imaginary part was smaller than that of the real part.

In the case of hemorrhagic tissue (clotted blood), the impedance of clotted blood was greater than that of fresh blood, which is in accordance with previous reports [33,45]. This may be related to blood coagulation, which is an extremely complex series of physiological processes. At the initial stage of coagulation, clotting factors, such as plasma protein fibrinogen and platelets, work immediately [33]. Platelets form plugs and the coagulation chain reaction forms fibrin strands. Fibrin formation is a complicated cascade including intrinsic and extrinsic pathways. After the formation of a fibrin mesh, blood coagulation is complete. During coagulation, blood changes from sol to gel state, which causes a change in molecular charge-state and effective charge mobility. In our study, the impedance of clotted blood remained constant at frequencies <10 kHz (Figure 4c), which is in agreement with the measurement of clotted blood reported by Burger et al. (20 Hz–5 kHz) [46]. Dowrick et al. used a two-electrode technique to measure the clotted blood obtained from a spontaneous coagulation process at 4 °C [34]; the effect of electrode impedance was compensated by subtracting the results from two sets of data. They found that the change in impedance was up to 10% below 250 Hz. The difference between our results and the results of Dawrick et al. may be explained by two factors. First, the two-electrode technique severely impacts the measured results because of electrode and contact impedance, especially at low frequencies (<1 kHz) [37]. Second, the sources of the blood samples are different. In the study by Dowrick et al., the clotted blood formed as the result of spontaneous coagulation at 4 °C, whereas the clotted blood in our study was obtained from a hemorrhagic animal model. Two different mechanisms of blood clotting under two conditions may produce different outcomes.

In our study, in order to control measurement conditions to ensure the accuracy of measurement, we used an infant incubator to maintain the temperature at 37 °C, with 60% humidity. Because we measured the tissue impedance within 15 min after animal death, there may be a difference between our measured results and the actual values of in vivo impedance before the sacrifice of the animal. However, the hematoxylin and eosin-stained images of normal brain tissues clearly show that the cells of normal white matter, as well as gray matter were arranged neatly and tightly. Furthermore, there are no morphological changes in normal tissue cells as in ischemic tissue cells (Figure 9). Therefore, we infer that this difference might exist, but should be small, and our measured results are reliable.

### 4.2. Helpful Information on Stroke Detection with MFEIT

When detecting stroke, the first step is to differentiate stroke lesions from normal brain tissue. In Figure 5, the difference in conductivity between ischemic tissue and white matter slowly changed with frequency, approximately 20% within the whole frequency range, while the difference in permittivity largely changed with frequency, approximately 50%. The difference in conductivity between ischemic tissue and gray matter remained at 20% over the whole frequency range, while the difference in permittivity remained at 10%. Thus, to differentiate ischemic tissue from normal tissue, the whole frequency range should be used. The difference in both conductivity and permittivity between hemorrhagic and normal tissue was greater at high frequencies (>100 kHz). For example, the difference in conductivity between hemorrhagic tissue and gray matter reached up to 50% and the difference in permittivity was up to three-times greater. Thus, to discriminate hemorrhagic tissue from normal tissue, the high-frequency range (>100 kHz) should be adopted.

The second step is to discriminate ischemic from hemorrhagic stroke. Figure 6 shows that the ratio of change in conductivity and permittivity of ischemic tissue at the low-frequency range (10 Hz–1 kHz; 7%) was significantly greater than in hemorrhagic tissue (0.5%). At the middle- (1 kHz–100 kHz) and high- (100 kHz–1 MHz) frequency range, the ratio of change in the permittivity of hemorrhagic tissue (16- and four-times, respectively) was significantly greater than in ischemic tissue (<2 times). Thus, the low- and middle-frequency range may be the optimal frequency needed to discriminate between the two types of stroke.

In this study, we established a PLS-DA model using conductivity and permittivity spectra to discriminate between different types of brain tissue. As shown by the PCA score plot, all measurements fell within their own unique tissue-specific area. Our regression analysis also showed that each tissue could be clearly separated. This is a good indication that the impedance spectrum of each tissue (gray matter, white matter, hemorrhagic and ischemic tissue) is unique and specific. In practice, stroke detection with MFEIT is very complicated. The boundary voltages always result from both normal head tissues and stroke lesions other than the latter alone, so PLS-DA would hardly be an appropriate method for stroke detection. However, based on this analysis, the specific impedance spectrum of each tissue over the whole frequency range may provide helpful information for stroke detection.

## 5. Conclusions

In this study, we established hemorrhagic and ischemic models and comprehensively measured and compared the impedance spectra of gray matter, white matter, ischemic and hemorrhagic tissue within 15 min after animal death using frequencies from 10 Hz–1 MHz. The results showed that the impedance spectra of stroke lesions obviously differed from normal brain tissue (the conductivity difference between normal brain tissue and stroke lesions was >10% within the 10 Hz–1 MHz range); the ratio of change in impedance in ischemic and hemorrhagic tissue with regard to frequency was distinct (within 100 kHz, the impedance spectrum of hemorrhagic tissue was predominately uniform, while a near linear change appeared in the impedance spectrum of ischemic tissue); and the results of the PLS-DA showed that the tissue impedance spectra could be used to discriminate the different types of tissue. These findings further validate the feasibility of detecting stroke with MFEIT by means of tissue impedance spectra in animal experiments and provide support for further study of stroke detection with MFEIT.
